# Assessment of attitudes towards future implementation of the “Surgical Risk Preoperative Assessment System” (SURPAS) tool: a pilot survey among patients, surgeons, and hospital administrators

**DOI:** 10.1186/s13037-018-0159-z

**Published:** 2018-06-04

**Authors:** Anne Lambert-Kerzner, Kelsey Lynett Ford, Karl E. Hammermeister, William G. Henderson, Michael R. Bronsert, Robert A. Meguid

**Affiliations:** 1 Surgical Outcomes and Applied Research program, University of Colorado School of Medicine, Aurora, CO USA; 2 Colorado School of Public Health, University of Colorado, Aurora, CO USA; 3 Department of Veterans Affairs Medical Center, VA Eastern Colorado Health Care System, Denver, CO USA; 4 Adult and Child Center for Health Outcomes Research and Delivery Science, University of Colorado School of Medicine, Aurora, CO USA; 5 Division of Cardiology, Department of Medicine, University of Colorado School of Medicine, Aurora, CO USA; 6 Department of Biostatistics and Informatics, Colorado School of Public Health, Aurora, CO USA; 7 Division of Cardiothoracic Surgery, Department of Surgery, University of Colorado Denver, Anschutz Medical Campus12631 E. 17th Avenue, C-310, Aurora, CO 80045 USA

**Keywords:** Surgical risk preoperative assessment, Qualitative methods

## Abstract

**Background:**

Risk assessment in surgery is essential to guide treatment decisions but is highly variable in practice. Providing formal preoperative risk assessment to surgical teams and patients may optimize understanding of risk. Implementation of the Surgical Risk Preoperative Assessment System (SURPAS), an innovative real time, universal, preoperative tool providing individualized risk assessment, may enhance informed consent and reduce adverse outcomes. To ensure optimal development and implementation of SURPAS we performed an in-depth pre-implementation evaluation of SURPAS at an academic tertiary referral center in Colorado.

**Methods:**

Four focus groups with 24 patients, three focus groups with 29 surgical providers and clinic administrators, and five individual interviews with administrative officials were conducted to elicit their perspectives about the development and implementation of SURPAS. Qualitative data collection and analyses, utilizing a Matrix Analysis approach were used to explore insights regarding SURPAS.

**Results:**

Participants were positive about SURPAS and provided suggestions to improve and address concerns regarding it. For healthcare personnel three major themes emerged: 1) *The SURPAS tool* - Important work especially for high risk patients, yet not a substitute for clinical judgment; 2) *Benefits of SURPAS to the risk assessment process* - Improves the processes, enhances patients’ participation in shared decision-making process, and creates a permanent record; and 3) *Facilitators and barriers of implementation of SURPAS* - Easy to incorporate into clinical practice in spite of surgical providers’ resistance to adoption of new technology. For patients three major themes emerged: 1) *Past experience of preoperative risk assessment discussions* – Patients were not made aware of possible complications that occurred; 2) *The SURPAS tool* - All patients liked SURPAS and believed having printed material would be useful to guide discussions and facilitate remembering conversations with the providers; and 3) *Potential concerns with having risk assessment information* – Patients were mixed in deciding to have an operation with high risks.

**Conclusions:**

Systematically capturing data from the beginning of the implementation process from key stakeholders (patients, surgical providers, clinical staff, and administrators) that includes adaptations to the tool and implementation process will help to inform pragmatic approaches for implementing the SURPAS tool in various settings, scaling-up, and sustaining it.

## Background

Perioperative complications from major surgical procedures occur in approximately 13% of patients and all-cause mortality in 1.4% of patients within 30 days after surgery, based on the American College of Surgeons (ACS) National Surgical Quality Improvement Program (NSQIP) dataset. These include infectious, cardiac, bleeding, renal, pulmonary, venous thromboembolic, and neurological complications, and death [1]. In addition to the detrimental impact that these adverse occurrences have on patients’ length and quality of life [2], healthcare costs of hospitalizations for patients experiencing perioperative complications can be up to five times that of patients without complications [3, 4]. Reduction of these complications is of great importance to patients, their families, surgical providers, healthcare payers, and society.

Identifying patients preoperatively who may have higher risks of complications may improve surgical care [5]. Presently, preoperative risk assessment of postoperative complications is typically based on accepted or previously reported values, and subjective assessment of individual patient comorbidities by providers, which may vary widely in accuracy [5, 6]. Formal risk assessment tools exist, many based on ACS NSQIP data, but are not widely used perhaps because they are seldom easy to use or not integrated into clinical workflow, i.e., the electronic health record (EHR) [5, 6]. We argue that the next generation of preoperative risk assessment tools needs to be quick and easy to use, integrated into the EHR, provide reliable and meaningful estimates of risk, encompass many different types of surgery and complications, be based on readily available preoperative data, and be updated periodically [6].

**Fig. 1:**
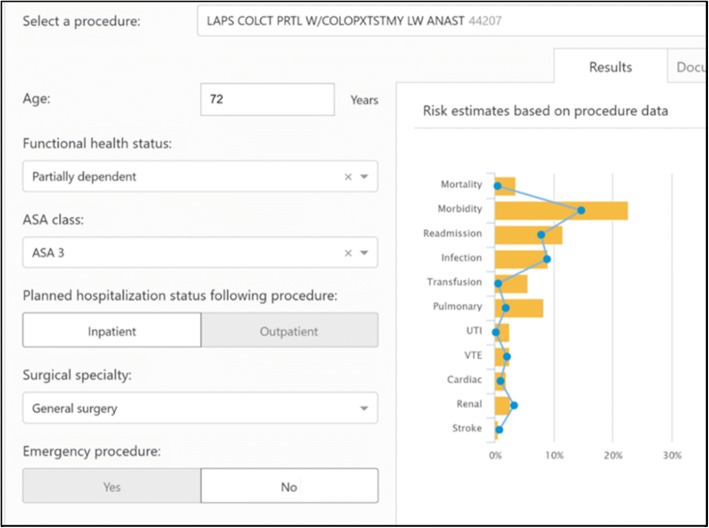
Example of SURPAS CDS output for sharing calculated patient risk of postoperative outcomes with the provider

Consequently, we are developing the Surgical Risk Preoperative Assessment System (SURPAS) clinical decision support (CDS) system. SURPAS is based on ACS NSQIP data, which has an exclusion criterion of patients under the age of 18 years. The design and statistical methodologies of SURPAS have been described previously [1, 5, 7]. This innovative tool provides individualized preoperative risk assessment for eight different 30-day postoperative adverse outcomes: mortality, overall morbidity, and six complication clusters (infectious, transfusion and cardiac, renal, pulmonary, venous thromboembolic, and neurological complications). SURPAS provides accurate risk assessments based on eight preoperatively available predictor variables, four of which are operative characteristics (work Relative Value Unit, inpatient/outpatient operation, primary surgeon specialty, and emergency operation status) and four of which are patient characteristics (American Society of Anesthesiology physical status classification (ASA class), functional health status, age, and sepsis within 48 h of surgery). Age and primary surgeon specialty are prepopulated from the local EHR and the clinician enters the remaining six variables into the SURPAS EHR interface. Upon completion of data input, a screen report is generated [Fig. 1] providing a graphical display and table with the patient’s individual calculated risk for each postoperative adverse outcome compared to the average patient undergoing the same operation.

Providing formal preoperative risk assessment to surgical teams and patients may optimize understanding of risk and perioperative care of surgical procedures for both elective and emergency operations [8]. As part of the implementation of SURPAS, we greatly value the input of stakeholders: patients, surgical providers, and administrators concerned with the delivery of surgical care [8]. Obtaining end-user opinions and perspectives throughout the development and implementation of SURPAS is based on the realist philosophy of Pawson [9] and the Medical Research Council [10, 11], who argue that evaluation needs to identify “what works in which circumstances and for whom?” not just “does it work?” [9–11] This study reports on the pre-implementation evaluation of SURPAS to optimize its development and implementation utilizing a qualitative methodology with focus groups and individual interviews of patients, surgical providers, and administrators.

## Methods

### Data collection

Surgical patients who underwent an operation within the previous year were recruited at an academic tertiary referral center in Colorado via a letter of introduction about the study. One hundred letters were sent out in three waves. Thirty two patients responded with interest and the study staff called each patient to schedule a focus group. The dates for the three focus groups were based on common availability of most patients. A total of 18 patients attended one of the three focus groups. All patients who attended one of the original focus groups were invited to attend a follow-up focus group approximately 1 year later. Of the 18 invited, six attended the follow up focus group. All patients received $75 for their participation. A convenience sample of surgical provider and clinical researcher participants was recruited from our institution’s Surgical Outcomes and Applied Research program to participate in two focus groups. A follow-up provider/clinical researcher focus group was held approximately 1 year later. Individual interviews were performed with administrative officials. Postcard informed consent was obtained at the time of focus groups and interviews. This study was approved by our institution’s institutional review board including HIPAA compliance.

Interviews and focus groups were conducted by Masters and PhD-educated members of the team trained in qualitative research (ALK, KLF). All participants viewed a standardized SURPAS presentation followed by discussions about the tool and the provider and patient data displays [12]. Semi-structured questions were designed to elicit opinions about SURPAS, suggestions to improve the tool, and barriers and facilitators to its implementation. During patient focus groups, participants were asked to describe their past experience with surgical risk assessments. The 60–90 min focus groups and interviews were audio-recorded and transcribed verbatim by a transcription service.

### Data analysis

Inductive and deductive analytical strategies drawing primarily on matrix and reflexive analysis were used to analyze the qualitative data [13, 14]. A matrix analysis was created using a priori codes and codes which emerged from the participant responses. The validity and accuracy/reliability of the early codes were established by two trained qualitative analysts (ALK, KLF), who analyzed the initial transcripts, coming to consensus, thus defining the initial codebook [13–16]. Subsequent transcripts were analyzed and new codes emerging from the data were added throughout analysis. Analysis of the codes resulted in the emergence of themes. The consistency of coding/interpretation was reviewed by all co-authors at monthly group meetings and discrepancies were addressed through discussion and consensus. This process continued until thematic saturation (not hearing any new information) was achieved [13–16]. All analyses and findings were integrated and documented with an audit trail [13–16]. Illustrative quotes were selected by consensus of all members of the analytic team to ensure representativeness across interviews.

## Results

We report the results of the two phases of the study, first the focus groups of and interviews with clinical providers and administrators, and then the focus groups with patients.

### I. Clinical provider and administrator focus groups and administrative official interviews

Two focus groups of clinicians included 18 participants (10 surgeons, 1 internist, 2 anesthesiologists, 1 biostatistician, 2 medical students, and 2 administrative officials). Five additional administrative officials were interviewed for their perspectives on SURPAS. Baseline demographics are provided in Table 1. A follow-up focus group with 11 surgeons was held to assess their opinions of the SURPAS tool 1 year later, after further development and refinement of the tool.

**Table 1:** Provider and administrator demographics

Roles	Mean Age in Years	Number of Females (%)	Educational Attainment (*N*)
Clinic Administrators (*N* = 2)	Unknown	*N* = 1 (50%)	MHA^a^ (*N* = 1) BS^b^ (*N* = 1)
Providers (*N* = 13)			
Surgeon (*N* = 10)	48	*N* = 3 (30%)	MD^c^ (*N* = 7) MD^c^, PhD^d^ (*N* = 1) MD^c^, MHS^e^ (*N* = 1) MD^c^, MBA^f^ (*N* = 1)
Anesthesiologist (*N* = 2)	53	*N* = 1 (50%)	MD^c^ (*N* = 2)
Internist (*N* = 1)	Unknown	*N* = 1 (100%)	MD^c^, PhD^d^ (*N* = 1)
Clinical Researcher (*N* = 1)	45	*N* = 0 (0%)	MS^g^ (*N* = 1)
Medical Student (*N* = 2)	23.5	*N* = 0 (0%)	BS^b^ (*N* = 2)
Administrative Official (*N* = 5)	Unknown	*N* = 1 (20%)	MD^c^ (*N* = 4) MBA^f^ (*N* = 1)

^a^MHA - Master of Health Administration

^b^BS - Bachelor of Science

^c^MD - Doctor of Medicine

^d^PHD - Doctor of Philosophy

^e^MHS - Master of Health Science

^f^MBA – Master of Business Administration

^g^MS - Master of Science

Overall, the clinician and administrative participants were positive about SURPAS. Three major themes emerged, discussed in depth below: 1) Overall opinions of, suggestions for, and concerns with the SURPAS tool; 2) Benefits for surgeons, patients, and the healthcare system of the tool to the risk assessment process; and 3) Facilitators and barriers of implementation of the tool in clinic [Table 2]. Individual quotes from themes are provided in Table 3.

**Table 2:** Provider, administrator, and clinical researcher themes

Overall Opinions of, Suggestions for, and Concerns with SURPAS Tool	Facilitators and Barriers of Implementation of SURPAS Tool in Clinic/System	Benefits for Surgeons, Patients, and Healthcare System of SURPAS Tool
Overall positive feedback	Easy to incorporate intoclinical practice	Improves consent process
Patient-centered and supportsshared decision making	Pilot studies necessary to support implementation	Can be used to mitigate certain adverse outcomes
Very important work – improves risk assessment	Build on the early wins	Improves patient education
Usability and user-interface suggestions	Market the value to the end user	Enhances patient participation and satisfaction
Not a substitute for clinical judgment	Be adaptable to different clinical workflows	Part of permanent record to support patients, providers, and healthcare systems
Accuracy concerns	Potential resistance to change	Collaborative approach to care when utilized by all staff
Concerns about exposure to litigation	Level of training required to operate	Tailored risk assessment to patients

**Table 3:** Provider and administrative quotes

Theme	Quote
The overall opinion of the SURPAS tool	*“If I knew that if I did this [used SURPAS] and that it showed my patient is much higher risk than I had previously thought,[and] if there’s something I can do that’s going to lower that, then that’s a motivator.”* - Surgeon
The overall opinion of the SURPAS tool	*“I think the things that I like about it [SURPAS] is it takes something that’s incredibly challenging to do, both from the physician side and from the patient side, one, to be able to tell the story, and two, is for a patient to be able to understand the story. It makes it simple on both ends so it’s not a lot of work for the physician, and it’s also not a lot of work for the patient.”* - Administrative Official
Implementation of the tool into a system/clinic environment	A surgeon felt it should be used, *“When you’re actually really taking a serious look at all the data, tests, and the patient. I think to do it too far in advance, you miss a lot of the details in terms of decision-making.”* *“From an administrative standpoint, I’m interested in it for two reasons. One is we obviously want our patients to understand what they’re getting themselves into and be fully informed. This is gonna allow that to be much more likely, and that’s obviously what we want. I’ll be even more interested 5 years from now when you have the interventions because [we can] come back and say we now know that if you can quit smoking, your risk of wound infection goes down, your pulmonary outcomes will improve by blank percentage,* et cetera*.”* - Administrative Official
Benefits to surgeons of the SURPAS risk assessment process	*“This would change my decision making process; potentially thinking twice about a procedure depending on specific significant risks.”* - Surgeon
Benefits to patients of the SURPAS risk assessment process	One provider described using SURPAS in a difficult decision where surgery is not recommended due to anticipated high risk of mortality and morbidity: *“This [surgery] really isn’t going to make much of a difference in the outcome and we have a family wanting us to do everything.”* *“I think with our challenges of the population that we serve, very broad base,extremely diverse, with 120 languages that are spoken around just the hospital itself and the translation issues that we have, first of all, the interface, I think, makes a lot of sense, just showing simply, “Here’s your risk out of 100 people of what’s going to potentially happen. Now, that probably needs to be translated in other languages if we’re gonna be completely effective at just starting there, to have a way to have a conversation, even with a translator present”* - Administrative Official

#### Overall opinions of, suggestions for, and concerns with the SURPAS tool

- Overall opinion of SURPAS:

Participants expressed that utilization of databases and streamlining of calculations to assess risk is important work. They shared that SURPAS would provide individualized risk assessment for specific operations, and that it would be useful to know estimates of risk for the procedure, especially for high risk patients. SURPAS may provide a process of care to facilitate risk assessment and lower the risk of surgical complications via implementation of bundles of care for high risk patients.

Most providers believed the tool would facilitate patient-centered care and the shared decision making process with patients and families. SURPAS could be extraordinarily helpful by strengthening the discussion of perioperative risks when addressing specific complications, by offering concrete discussion points for each patient.

Administrative officials indicated that it is important for patients to fully understand and have clear expectations about the operation, and that they may have the opportunity to improve their outcomes by choosing to adhere to prescribed preventive measures. They also believed SURPAS would improve the provider experience, as opposed to just adding work for providers, and would help to create safer delivery of healthcare.

- Concerns with SURPAS:

Some concerns over its use emerged. Surgeons articulated that SURPAS was not a substitute for clinical judgment and providers were still responsible for determining what about the patient was contributing to their risk. A few participants were concerned that if some patients are presented with high risk of adverse outcomes, especially if they are higher than patients expect, they would not want to proceed with surgery, or would go elsewhere for care by a different surgeon.

Medical-legal issues were raised by surgeons and administrators, and included reactions of patients when the assessed risk was low, yet the patient ultimately experienced a complication. Some surgeons were concerned about potential reactions from insurance companies such as challenging plans for surgery because the patient had a high risk estimate. Finally, some participants were concerned with the subjectivity of the ASA class (one of the predictors used for risk calculation) as determined by surgeons, especially in complex patients.

- Suggestions to improve SURPAS:

Participants offered the following suggestions to improve SURPAS: 1) Split the “cardiac/transfusion” complication cluster into separate “cardiac” and “transfusion” complication. Separate “urinary tract infection” (UTI) from the “infectious” complication cluster [superficial surgical site infection (SSI), deep incisional SSI, organ/space SSI, wound disruption, and sepsis] because they are addressed via different processes of care. 2) Provide definitions of the ASA class, emergency/elective operation status, and functional health status on the input screen; 3) Avoid providing default values for variables as they may bias data collection; 4) Provide drop-down menus for the input of predictor variables; 5) Provide a drop-down list of Current Procedural Terminology (CPT) codes and names for operations frequently performed by the provider; 6) Perform periodic updates to the risk models; 7) Prevent the burden on the provider from being increased by making SURPAS “click neutral”, meaning it should not increase data entry into the EHR; and 8) Provide audit and feedback of postoperative complications.

#### Benefits for surgeons, patients, and the healthcare system of SURPAS to the risk assessment process

- To surgeons:

Most participants believed the SURPAS tool would improve preoperative risk assessment processes, provide documentation of the risk information and discussion, and improve appropriate discussions with the patient, caregiver, and family guiding the informed consent process. Use of SURPAS could replace the practice of providing generalized risks with personalized risks, provide data to support surgeons’ opinions, give help to providers in identifying high risk patients and possibly mitigating some adverse outcomes via preoperative interventions, or could help institute patient-specific postoperative interventions to improve patients’ clinical courses and outcomes.

- To patients:

Providers thought SURPAS would enhance patients’ participation in the shared decision-making process, and support better management of expectations with improved patient education. SURPAS was considered particularly valuable for assessment of high-risk patients where the patient and family are assessing quality of life outcomes. One provider proposed that SURPAS would potentially support communication across multiple languages and cultures.

- To the healthcare system:

SURPAS provided a document in the permanent patient record indicating that a risk assessment was performed and an informed conversation about risk occurred. This could be referenced by other clinical staff, supporting multidisciplinary collaboration, which may decrease the likelihood of critical omissions in patient care, thus supporting patients, providers, and the healthcare system.

#### Facilitators and Barriers of Implementation of SURPAS in clinic

- Facilitators to implementation:

Most participants thought the tool would be easy to incorporate into clinical practice and facilitated by gaining buy-in from the local EHR team and end-users. Differing opinions were expressed about making use of the tool mandatory. Due to the wide variation in workflow in surgical clinics, pre-implementation site visits with clinicians and clinic managers should be performed. Pilot studies were suggested to assess best practice in the clinical environments.

Administrative officials focused on strategies to facilitate the uptake of the SURPAS tool. These included: to clearly define and assign specific responsibilities to key stakeholders, including implementation and technical support for SURPAS; and that providers need to be incentivized with factors such as early *“wins,”* including the ease of use with limited data entry, automated documentation, increased knowledge to guide decisions making, and improved facilitation of patient communication. The next steps included “*marketing*” the value to the end user to *“generate buzz”*, use of feedback mechanisms from providers to improve the tool and the implementation process, and identifying local champions to support deployment and enhance self-sustained use of SURPAS.

Use of audit and feedback, involving collecting clinical performance data over a specified time period and providing it to clinicians and administrators to monitor, evaluate, and if needed to modify provider behavior, was suggested. SURPAS could be tied to strategic goals of the healthcare system, including innovation, safety, access, growth, and patient-centeredness. The final suggestion included providing scripted answers to frequently asked questions to facilitate the implementation of SURPAS and foster collaborative discussions with patients about their surgical risk and decision making.

- Barriers to implementation:

Key barriers to use of SURPAS were identified as: (1) Surgical providers’ resistance to adoption of new technology; (2) Change in workflow resulting from integration of SURPAS into their preoperative assessment process; and (3) Individual providers may not agree that the projected risk applies to their patients..

Some providers thought that the effectiveness of SURPAS in lowering complications must be demonstrated before broad implementation could occur and that inter-rater reliability studies may be warranted to confirm that surgeons can accurately determine ASA class. Several providers believed the greatest obstacle to acceptance was the accurate representation of the intended operation based on selection of only one CPT code, as it may not accurately predict risk for more complex surgeries defined by multiple CPT codes.

### II. Surgical patients focus groups

Three focus groups, totaling 18 surgical patients, were convened to elicit patient perspectives of SURPAS. Baseline demographics are provided in Table 4. Three overarching themes emerged: 1) Past experience of preoperative risk assessment discussions; 2) The SURPAS tool; and 3) Potential concerns with having risk assessment information [Table 5]. Individual quotes for themes are provided in Table 6. A follow-up focus group with six participants from the original focus groups was held to assess their opinions of the refined SURPAS tool.

**Table 4:** Patients demographics

Patients (*N* = 18)	Number	Percent
Age (years) (*N* = 18)		
30–39	2	11.1%
40–49	6	33.3%
50–59	4	22.2%
60–69	4	22.2%
70+	2	11.1%
Gender (*N* = 18)		
Female	13	72.2%
Types of Surgery (*N* = 21)		
Orthopedic		
Hip Replacement	2	9.5%
Knee Replacement	2	9.5%
Rotator Cuff Repair	1	4.8%
Elbow Surgery	1	4.8%
General Surgery		
Appendectomy	1	4.8%
Cholecystectomy	1	4.8%
Mastectomy	2	9.5%
Breast Reconstruction	1	4.8%
Urology		
Radical Cystectomy	1	4.8%
Bladder Reconstruction	1	4.8%
Cardiothoracic Surgery		
Lung Lobectomy	1	4.8%
Pleurodesis	1	4.8%
Mitral Valve Replacement	1	4.8%
Gynecologic Surgery		
Total Abdominal Hysterectomy	1	4.8%
Salpingoophorectomy	1	4.8%
Vascular		
Hemodialysis Fistula Creation	1	4.8%
Otolaryngology		
Salivary Gland Removal	1	4.8%
Other		
Unknown	1	4.8%s

**Table 5:** Patients themes

Issues that Emerged from Past Experience of Preoperative Risk Assessment Discussions	The Overall Opinion of the SURPAS tool	Potential Concerns with Having Risk Assessment Information
Some patients needed more information than others	All patients liked SURPAS. Patients wanted to have a visual display of the risk to take home; They preferred the pictogram of 100 patients	Patients had mixed reactions to the question, “Would you still have the operation if your risk is high?”
A feeling of being overwhelmed with the information - Not being able to remember details after the clinic visit	They believed it would be informative for relaying individual surgical risks	An additional concern existed over the scenario where the estimated risks were low but the patient still suffered a complication
Information should be given to caregivers or family members	SURPAS facilitates understanding of their individual risk of complications, compared to an average risk

**Table 6:** Quotes from patients

Theme	Quote
Past experience of preoperative risk assessment discussions	*“You’re always told [the] risks. It’s verbalized. All this stuff is being thrown at you, the surgery, recovery, what’s gonna—all this stuff. You don’t necessarily always remember.”* *“I didn’t have family with me at the time, so when [the patient’s family] said “Well, what did they say?” and I didn’t hear a word after, “You need a total abdominal hysterectomy.”*
Issues that emerged from past experience of preoperative risk assessment discussions	For one patient, the emotional trauma was not explained as well as it could have been: *“I suffered from horrible depression–dark depression for a couple of months after the surgery that really impeded—I didn’t even want to get out of bed or walk or anything. I mean my mental state of mind, it went upside down.”*
The overall opinion of the SURPAS tool	*“This would make me as a patient pay more attention to this type of procedure, what are [the] risks?”* SURPAS *“...helps the patient feel more confident that the provider is listening to them, understanding their bodies, and it also puts some ownership … back on the patient.”* *“If your risk is more, I think it really will make you consider more carefully the benefits of the surgery as opposed to the problems that you could end up with. I can’t see that it would do any harm.”*
Opinion of SURPAS tool visual aids for patients/Preferred display of risks	*“...emotionally a wonderful piece of information. Knowing that I had even an increased risk or a decreased risk* versus *a national average, I think, would be also very helpful just as a comparison.*
Potential concerns with being provided risk assessment information	A patient worried that, *“People get caught up on the numbers. Percentages are great. People want to see that, especially when they’re individualized compared to the population in general... But, if a procedure doesn’t go well, if there’s a complication... “You told me [my risk] was… one [percent].” Now you’ve got a disgruntled patient. Your customer service ratings are going to go down. Litigation, try to go that route over a number getting hung up could be a little bit of a downside with nice personalized numbers as well.”*

#### Past patient experience of preoperative risk assessment discussions

Patients shared the complications which occurred to them and believed it would have been helpful to be aware of the possibility of these complications. One patient reported that a conversation with the surgeon about the patient’s prior surgical complications was not taken seriously, with the patient ultimately experiencing the same complication again. Another patient experienced debilitating postoperative depression and felt the possible emotional trauma from surgery was not explained well and suggested incorporating a risk for postoperative adverse psychological effects.

Some patients reported that risks of complications were not explained to the extent they desired. They felt overwhelmed with the risk information, not being able to remember details after the clinic visit. They believed risk information should also be provided to caregivers or family members, and wanted documents of risk information to take home with them before surgery. Patients were concerned about occurrence of postoperative complications and their subsequent management and wanted to know how and by whom complications will be managed.

#### The SURPAS tool

- Patients’ overall opinion of the tool:

All patients liked SURPAS and believed it would be informative for relaying risks of the planned operation and facilitate the patients’ understanding of their individual risk of complications. They felt SURPAS would individualize the surgical risk assessment process. Patients suggested that having printed material on risk of complications before surgery would be useful to guide discussions with family and caregivers after the preoperative clinic encounter, and would help them remember more details of the conversation with the surgical provider. Patients suggested we provide the average risk of the same procedure so they could see how they do in comparison. Patients were interested in receiving information on risk of discharge to a facility other than home and unplanned readmission as well as long term functional outcomes. These are additional outcomes that could be added to the SURPAS tool.

**Fig. 2:**
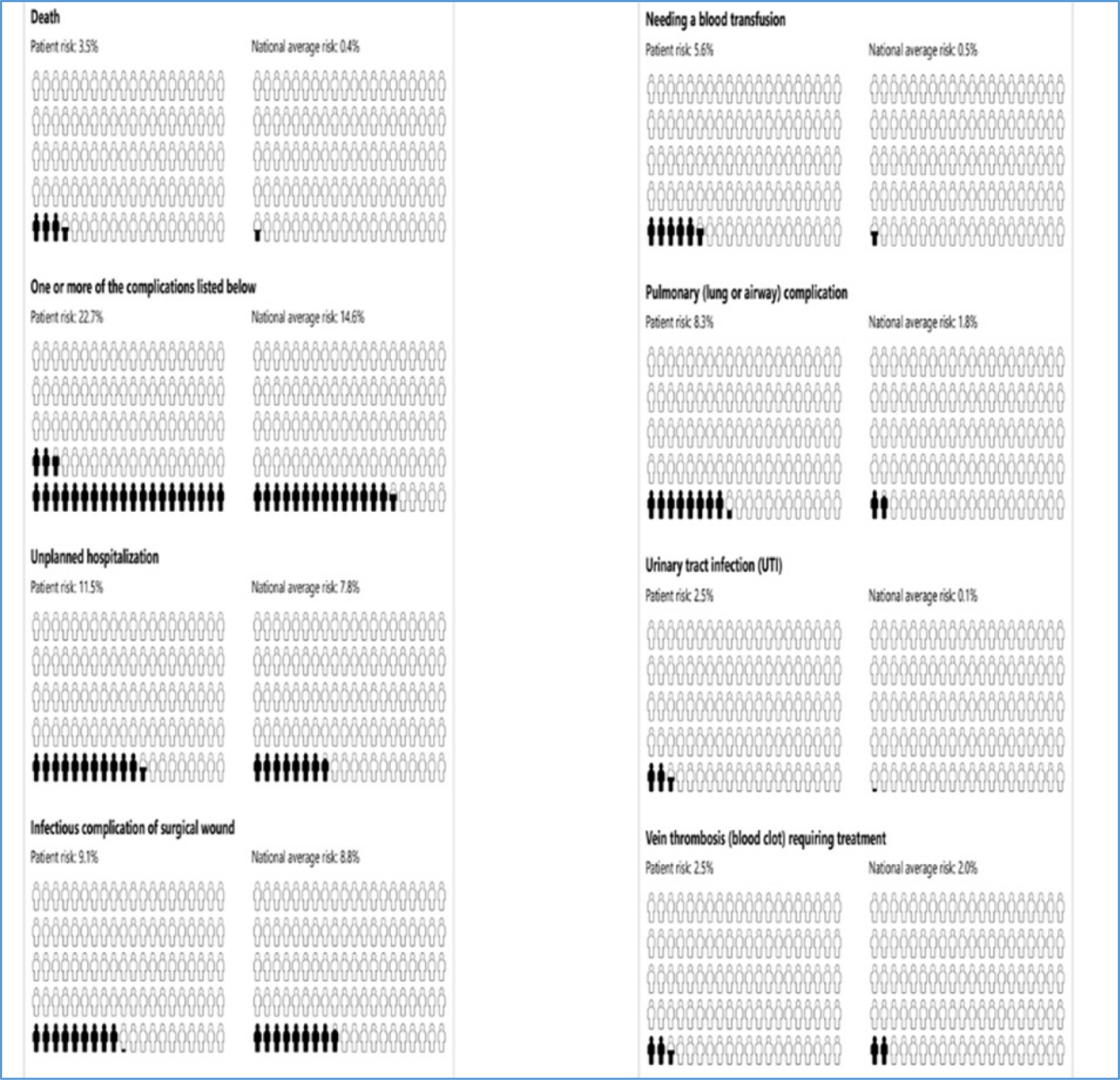
Pictograph example of SURPAS CDS output for sharing calculated patient risk of nine postoperative outcomes with the patient

- Opinion of SURPAS patient visualization and preferred display of risks:

Six options to visually display the risk to patients were presented: pictographs, bar graphs, pie charts, clock graphs, spark plug displays, and data in table format [12]. Of the 18 patients, 13 liked the pictograph [Fig. 2] for representation of personal risks of each complication. The second most-preferred display was the bar graph of the patient’s risk and a superimposed line graph showing the average risk for all patients undergoing the same operation [Fig. 1], which we used to display risk to providers.

#### Patients’ concerns with having risk assessment information

Patients had mixed reactions in response to the question, “Would you still have the operation if your risk of complication was high?” Some patients indicated that they did not consider *not* having surgery due to the natural history of their disease. One patient said he would have to weigh the potential risks with the potential benefits of the operation. Another concern patients raised was over the scenario where the preoperative risk estimates were low but they still suffered a postoperative complication.

## Discussion

Individualized preoperative risk assessment of adverse outcomes for patients undergoing surgical procedures has the potential to improve the processes of informed consent and shared decision making, guide perioperative care, and ultimately reduce occurrence of postoperative complications [6]. SURPAS was designed as a user-friendly tool to provide accurate risk assessment based on eight preoperatively available predictor variables. Through engagement of stakeholders, including patients, surgeons, anesthesiologists, medical students, clinic administrators, administrative officials, and clinical researchers, we obtained feedback to improve SURPAS and facilitate its implementation process.

Participants believed that risk assessment is very important and that it would be useful to have individualized risk assessment, especially for high risk patients. SURPAS may improve processes of care to reduce complications, facilitate patient-provider interactions, and improve patient-centered care. Furthermore, providers believed that protocols for bundles of care could be incorporated for high risk patients to reduce occurrence of potential adverse outcomes. Concerns regarding medical-legal issues were discussed as well as ensuring SURPAS was not a substitute for clinical judgment. Providers desired more evidence to support the accuracy of the SURPAS risk estimates for complex operations involving multiple CPT codes. Valuable suggestions were provided to improve SURPAS’ use and utility in the EHR.

Administrative officials’ guidance to facilitate implementation was based on their experiences with implementation of innovations. They suggested seeking early wins, generating interest in use of SURPAS, and performing iterative improvements. The identification of local champions would support deployment and help SURPAS to become self-sustaining.

Insightful discussions revealed barriers to the implementation of SURPAS with surgical providers’ resistance to adoption identified as a significant barrier. SURPAS needs to improve the provider experience, as opposed to just adding work and consuming time. Its effectiveness must be demonstrated and its use should result in lower complications or it will not be incentivized. Suggestions to facilitate implementation included performing pilot studies and assessment of the reliability of data collection and entry by providers (e.g., ASA class estimation by surgeons).

Patients thought SURPAS facilitated a personal conversation with their surgeon regarding risk and provided an opportunity to ask questions. Patients were concerned about postoperative care of complications and wanted such conversations to be included in the preoperative care. Having a document to visualize the risks at a later date and to share with family was important to the patients. The pictograph presentation of the individualized risk assessments identified by most as the preferred format will be used for displaying risk.

Based on the input we received from participants, the following changes were made to SURPAS: 1) Transfusion was separated from the cardiac complication cluster; 2) UTI was separated from the infectious complication cluster; 3) Unplanned readmission and discharge destination were added to the risk prediction models; 4) A patient handout providing individual risk estimates compared to population averages was developed so that patients and providers may see when predicted individual patient risk is greater than or less than that of the general population; 5) Dropdown fields for independent variables were added to the provider computer interface; 6) Default values for independent variables were removed from the interface; and 7) Definitions for some of the independent variables and outcomes were provided.

CDS systems are, “any electronic system designed to aid directly in decision making, in which characteristics of individual patients are used to generate patient-specific assessments or recommendations that are then presented to clinicians for consideration.” [17]. Studies have shown the importance of obtaining end-user and stakeholder evaluations throughout the development and implementation of new CDS systems [17–22].

Consequently, our pre-implementation study design and findings are supported in the literature. Kaplan et al. and Schoen et al. utilized similar study designs to support successful development and implementation of CDS tools [17, 19, 21, 22]. This phase of the development and implementation of SURPAS has incorporated the collaboration with clinical experts and patients who have contributed insightful critiques culminating with actionable suggestions to improve SURPAS’ usefulness, usability, and its presentation to patients and providers. The iterative of end-user feedback will continue with the next phase of the trial implementation that will call for a mixed methods approach, utilizing quantitative data collection of clinical outcomes integrated with observational and qualitative data assessments of the implementation process in real world settings that will include in-person surveys and interviews with patients and providers who have used the SURPAS tool during the pre-operative risk assessment clinic visit.

Strengths of this study include the initial integration of a broad range of opinions to improve SURPAS and identify barriers to and facilitators of the implementation process. This iterative process provides insights that will guide future iterations of SURPAS. Potential limitations may include social desirability bias – i.e., participants responding in a certain way to please the interviewer – and that the qualitative data were collected at only one location.

Key stakeholders were supportive of improving the risk assessment process, identified specific concerns, and provided suggestions to improve SURPAS. These suggestions have led us to further refine the SURPAS tool in order to improve the likelihood of adoption by surgical providers, provide added utility to patients, and minimize disruption of workflow in the busy clinic while increasing value of the CDS tool.

## Conclusions

SURPAS has the potential to enhance the informed consent and shared decision-making processes, guide perioperative care, and ideally, ultimately reduce occurrence of postoperative complications. Systematically capturing data from key stakeholders from the beginning of the implementation process, including adaptations to the tool, will help to inform pragmatic approaches for implementing the SURPAS tool in various settings, scaling-up, and sustaining it.

## Abbreviations

ACS: American college of surgeons
ASA class: American society of anesthesiology physical status classification
CDS: Clinical decision support
CPT: Current procedural terminology
EHR: Electronic health record
NSQIP: National surgical quality improvement program
SURPAS: Surgical risk preoperative assessment system
UTI: Urinary tract infection

## References

[1] Meguid R, Bronsert MR, Juarez-Colunga E, Hammermeister K, Henderson WG (2016). Surgical risk preoperative assessment system (SURPAS): I. Parsimonious, clinically meaningful groups of postoperative complications by factor analysis. Ann Surg.

[2] Khuri SF, Henderson WG, DePalma RG, Mosca C, Healey NA, Kumbhani DJ (2005). Determinants of long-term survival after major surgery and the adverse effect of postoperative complications. Ann Surg.

[3] Dimick JB, Chen SL, Taheri PA, Henderson WG, Khuri SF, Campbell DA (2004). Hospital costs associated with surgical complications: a report from the private-sector National Surgical Quality Improvement Program. J Am Coll Surg.

[4] Vonlanthen R, Slankamenac K, Breitenstein S, Puhan MA, Muller MK, Hahnloser D (2011). The impact of complications on costs of major surgical procedures: a cost analysis of 1200 patients. Ann Surg.

[5] Meguid R, Bronsert MR, Juarez-Colunga E, Hammermeister K, Henderson WG (2016). SURPAS III: accurate preoperative prediction of 8 adverse outcomes using 8 predictor variables. Ann Surg.

[6] Hammermeister KE, Henderson WG, Bronsert MR, Juarez-Colunga E, Meguid RA (2016). Bringing quantitative risk assessment closer to the patient and surgeon. A novel approach to improve outcomes. Ann Surg.

[7] Meguid R, Bronsert MR, Juarez-Colunga E, Hammermeister K, Henderson WG (2016). SURPAS II: parsimonious risk models for postoperative adverse outcomes addressing need for laboratory variables and surgeon specialty-specific models. Ann Surg.

[8] Stacey D, Legare F, Lewis K, Barry MJ, Bennett CL, Eden KB, et al. Decision aids for people facing health treatment or screening decisions. Cochrane Database Syst Rev. 2017; 10.1002/14651858.CD001431.pub5. 10.1002/14651858.CD001431.pub5 PMC6478132 28402085

[9] Pawson R (2006). Evidence-based policy: a realist perspective.

[10] Craig P, Dieppe P, MacItyre S, Mitchie S, Nazareth I, Petticrew M (2008). Developing and evaluating complex interventions: the new Medical Research Council guidance. BMJ.

[11] Campbell N, Murray E, Darbyshire J, Emery J, Farmer A, Griffiths F (2007). Designing and evaluating complex interventions to improve health care. BMJ.

[12] Hawley ST, Zikmund-Fisher B, Ubel P, Jancovic A, Lucas T, Fagerlin A (2008). The impact of the format of graphical presentation on health-related knowledge and treatment choices. Patient Educ Couns.

[13] Miles MB, Huberman AM, Saldaña J (2014). Qualitative data analysis. A Methods Sourcebook.

[14] Averill JB (2002). Matrix analysis as a complementary analytic strategy in qualitative inquiry. Qual Health Res.

[15] Patton M (2002). Qualitative Research & Evaluation Methods.

[16] Tong A, Sainsbury P, Craig J (2007). Consolidated criteria for reporting qualitative research (COREQ): a 32-item checklist for interviews and focus groups. Int J Qual Health Care.

[17] Schoen DE, Glance DG, Thompson SC. Clinical decision support software for diabetic foot risk stratification: development and formative evaluation. J Foot Ankle Res. 2015; 10.1186/s13047-015-0128-z. 10.1186/s13047-015-0128-z PMC4676878 26692903

[18] Kawamoto K, Houlihan CA, Balas EA, Lobach DF. Improving clinical practice using clinical decision support systems: a systematic review of trials to identify features critical to success. BMJ. 2005; 10.1136/bmj.38398.500764.8F. 10.1136/bmj.38398.500764.8F PMC555881 15767266

[19] Kaplan B. Evaluating informatics applications-some alternative approaches: theory, social interactionism, and call for methodological pluralism. Int J Med Inform. 2001; 10.1016/S1386-5056(01)00184-8. 10.1016/s1386-5056(01)00184-8 11673101

[20] Catwell L, Sheikh A. Evaluating eHealth interventions: the need for continuous systemic evaluation. PLoS Med. 2009; 10.1371/journal.pmed.1000126. 10.1371/journal.pmed.1000126 PMC2719100 19688038

[21] Kaplan B, Duchon D. Combining qualitative and quantitative methods in information-systems research - a case-study. MIS Q. 1988; 10.2307/249133.

[22] Sim I, Gorman P, Greenes RA, Haynes RB, Kaplan B, Lehmann H (2001). Clinical decision support systems for the practice of evidence-based medicine. J Am Med Inform Assoc.

